# Transplantation of Encapsulated Pancreatic Islets as a Treatment for Patients with Type 1 Diabetes Mellitus

**DOI:** 10.1155/2014/429710

**Published:** 2014-01-30

**Authors:** Meirigeng Qi

**Affiliations:** ^1^Division of Transplantation/Department of Surgery, University of Illinois at Chicago, IL 60612, USA; ^2^Department of Diabetes and Metabolic Diseases Research, Beckman Research Institute of the City of Hope, 1500 E. Duarte Road, Duarte, CA 91010, USA

## Abstract

Encapsulation of pancreatic islets has been proposed and investigated for over three decades to improve islet transplantation outcomes and to eliminate the side effects of immunosuppressive medications. Of the numerous encapsulation systems developed in the past, microencapsulation have been studied most extensively so far. A wide variety of materials has been tested for microencapsulation in various animal models (including nonhuman primates or NHPs) and some materials were shown to induce immunoprotection to islet grafts without the need for chronic immunosuppression. Despite the initial success of microcapsules in NHP models, the combined use of islet transplantation (allograft) and microencapsulation has not yet been successful in clinical trials. This review consists of three sections: introduction to islet transplantation, transplantation of encapsulated pancreatic islets as a treatment for patients with type 1 diabetes mellitus (T1DM), and present challenges and future perspectives.

## 1. Introduction

### 1.1. Type 1 Diabetes and Its Treatment

Type 1 diabetes mellitus (T1DM), also known as insulin-dependent diabetes mellitus, is an autoimmune disease that causes a progressive destruction of the insulin-producing pancreatic *β* cells [1, 2]. As a result, patients require exogenous insulin to maintain normal blood glucose levels. In patients with T1DM, long-term hyperglycemia often causes complications such as nephropathy, neuropathy, and retinopathy. According to a report from the American Diabetes Association (ADA), there are nearly three million children and adults living with T1DM in the USA and millions of others affected worldwide [3]. Management of T1DM and other associated complications is burdensome to both individuals and to society as a whole.

Insulin injection is a common method to directly control blood glucose levels. However, intensive insulin therapy can induce more frequent episodes of hypoglycemic symptoms in certain populations of patients with T1DM [4, 5].

Whole pancreas transplantation, which has been conducted since 1966, is a therapeutic way of stopping the progression of diabetic complications without increasing the incidence of hypoglycemic events [6–10]. The Graft survival rate has been well maintained post-surgery, with a survival rate of 76% after one year and 62% after three years. Long-term normoglycemia under insulin independence has been achieved with a 5-year graft survival rate of 50–70% [11]. Unfortunately, this procedure, which is usually performed simultaneously with kidney transplantation, involves complicated surgical procedures and consequential complications. Major complications include graft thrombosis, graft pancreatitis, pancreatic fistulae, and pseudocyst formation [12].

**Figure 1:**
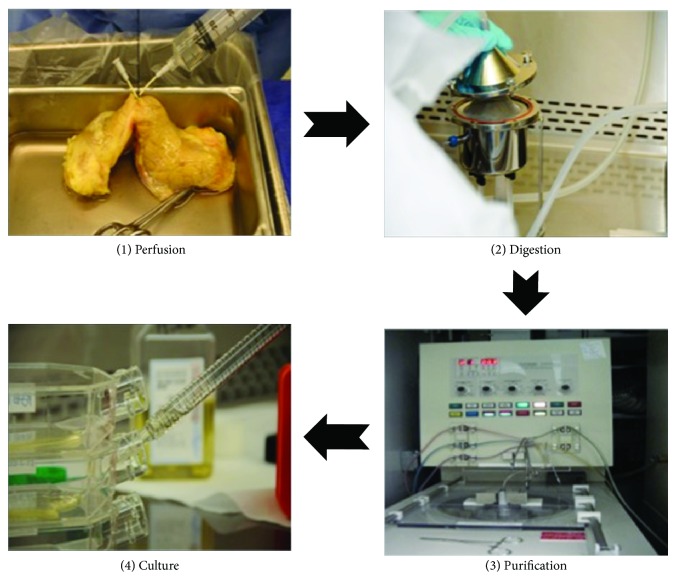
Human islet isolation procedure. (1) Pancreas perfused with enzymatic solution; (2) pancreatic tissue digested in Ricordi isolation chamber; (3) digested tissue purified in COBE 2991 cell separator; (4) purified islets cultured at 37°C/5% CO_2_.

Islet transplantation is considered as an improved way to cure T1DM in comparison with insulin injection and whole pancreas transplantation. Absence of insulin in patients with T1DM forces them to use exogenous insulin to maintain normal blood glucose, which can delay or prevent health complications. Theoretically, exogenous insulin can replace *β* cells in islets, but practically, the insulin injection cannot maintain stable blood glucose levels. Pancreatic islet transplantation is a procedure to selectively transplant the endocrine part of a whole pancreas (about 2% of the pancreas mass). In comparison with whole pancreas transplantation, islet transplantation can be conducted via a minimally invasive approach and is associated with minimal or no complications. The islets can be infused via a catheter that has percutaneous portal venous access [13]. Therefore, this procedure can be applied to a wider range of recipients. More importantly, the islet transplantation can provide glycemic control without exogenous insulin and risks of hypoglycemia. The first experimental islet transplantation was conducted in a rodent model in 1972; several years after this a whole pancreas transplantation was initiated in a human patient [14]. Generally speaking, clinical allogeneic islet transplantation involves four chronological steps: procurement of donor pancreas, isolation of pancreatic islets (Figure 1 and Table 1), assessment of isolated islets (Table 2), and transplantation of harvested islets and patient followup.

Although islet transplantation has been widely accepted in recent years, the protocol has not obtained a license and is not accepted as a standard clinical treatment. Currently, many islet transplantation centers are planning or initiating license applications for clinical allogeneic islet transplantation.

### 1.2. Limitation of Islet Transplantation and Initiation of Encapsulation

Although the field of islet transplantation has progressed rapidly, the long-term success of allogeneic islet transplantation remains questionable. Patients from the original Edmonton trial had an insulin-independence rate of approximately 10% at five years after transplantation [20]. This rate, based on a recent study, is as high as 50%, but the combination of an optimized immunosuppressive regimen and a sophisticated transplant center is required [21]. As discussed elsewhere, the reasons for long-term graft loss can be summarized into the two following categories.

*(i) Immunosuppression Associated Factors*. Islet recipients must take immunosuppressive medications to prevent allogeneic rejections. Any imperfect immunosuppressive protocol can lead to graft loss. But after long-term usage, even the optimized medications can be toxic to the transplanted islets directly or cause dysfunction of other organs [20]. In addition to the damage allogeneic rejection can cause to the transplanted islets, recurrence of autoimmune attacks on the transplanted islets has also drawn investigator's attention. Histological studies have shown that islet transplantation triggers recurrent autoimmune effects that can cause *β*-cell destruction [22, 23]. Another study has revealed that the presence of pretransplant autoreactivity could lead to strengthened autoimmune reactions targeting *β* cells [24].

*(ii) Nonimmunosuppressive Associated Factors.* Nonimmunosuppresive factors including insufficient islet mass and poor islet quality can cause the dysfunction of islets in the long term. Islets are transplanted through the portal system and engraft in the liver; this can cause islet graft loss by (1) instant blood-mediated inflammatory reaction (IBMIR) [24]; (2) hypoxia-related islet cell death [25]. It has been reported that approximately 60% of pancreatic islets are destroyed due to IBMIR after intraportal transplantation [26]. This reaction leads to the disruption of islets due to the activation of complement and coagulation systems [27, 28]. Tissue factor together with monocyte chemoattractant protein (MCP-1) and other inflammatory mediators cause the activation of coagulation and complement system. Poor clinical outcomes of islet transplantation are often associated with increased intensity of IBMIR [29]. In terms of hypoxia-related islet loss, the devascularization caused during the isolation, as well as the implantation of the islets into low oxygen tension within the liver, directly damages the islet cells [25]. The indirect cause of islet loss can be considered as the result of the activation of innate immune system by the hypoxia environment itself. Consequently, the release of inflammatory cytokines, such as tumor necrosis factor-*α* (TNF-*α*), interferon-*γ* (IFN-*γ*), and interleukin-1*β* (IL-1*β*), damages the islet graft [30].

With the above-mentioned limitations, the field of islet transplantation has been trying to find an alternative strategy to minimize the limitations for both donors and patients. Specifically, two avenues of research are being investigated. First, to find a possible method to decrease islet loss or provide an unlimited source of islet cells for transplantation. Second, to find an alternative approach to avoid the use of immunosuppressive medications. Immunoisolation of pancreatic islets, also known as encapsulation, not only allows for transplantation of cells without immunosuppression but also increases the chance of using cells from a nonhuman origin.

## 2. Transplantation of Encapsulated Pancreatic Islets as a Treatment for Patients with T1MD 

### 2.1. Overview of Encapsulation

Cell encapsulation technology is based on the concept of immunoisolation, which was originally presented by Prehn et al. from as early as 1954. Prehn et al. used a type of immunoisolation instrument called the diffusion chamber device [31]. In that study, the diffusion chamber device was used to prevent the homograft from provoking an immune reaction in the host. Later on this technology was used to protect transplanted cells, known as “artificial cells” [32–36]. Since islet cells can be isolated and transplanted successfully, the encapsulation technology was soon applied in the field of islet transplantation. Many types of encapsulation technologies have been investigated over the last three decades in different animals such as mice [37], rats [38], dogs [39, 40], and monkeys [41, 42]. These studies demonstrate the feasibility of restoring normoglycemia by implanting allo- and xenografts without immunosuppression. Furthermore, the studies reveal the inconsistency of transplantation outcomes due to differences in encapsulation strategy and in animal models. The studies also suggest that long-term graft survival might depend on enriched and consistent blood supply to the grafts. In the light of the experiences accumulated from the large amount of transplant studies performed in different animal models, scientists and clinicians attempted a trial involving encapsulated allogeneic islet transplantation in patients with T1DM [15, 17, 18, 43]. The following sections describe the encapsulation technologies, characterize insulin release from encapsulated islets, depict immunology and biocompatibility factors of the devices, outline the approach of local/short-term immunomodulation, report the trials of clinical encapsulated islet transplantation, and discuss alternative cell sources for encapsulation.

### 2.2. Cell Encapsulation Technology

**Figure 2:**
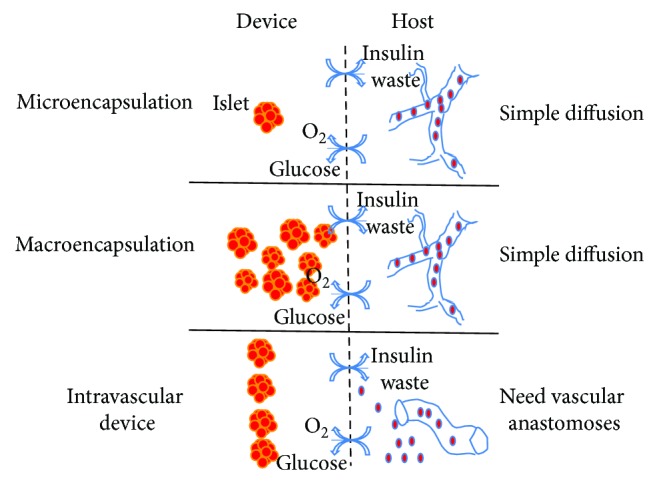
Schematic representation of immunoisolation device or bioartificial pancreas. They can be commonly separated into two categories, intravascular and extravascular devices. The latter can further be divided into macroencapsulation and microencapsulation devices. Intravascular and extravascular classifications are based on whether or not it is connected directly to the blood circulation. The macroencapsulation and microencapsulation classifications depend on whether it contains one or more islets in the device.

Encapsulation technology provides the means for islet cell survival in the absence of immunosuppressive drugs. The principle of encapsulation is that transplanted cells are contained within an artificial compartment separated from the immune system by a semipermeable membrane. The capsule should protect the cells from potential damage caused by antibodies, complement proteins, and immune cells. Therefore, the capsule is often referred to as an “immunoisolation device.” As well as the protective mechanism provided by the capsules, islet cells within the capsules can also release insulin to control blood glucose levels, since this membrane enables small molecules to diffuse in (glucose, oxygen, and nutrients) and out (metabolic wastes). Thus, the encapsulation system is also regarded as a “bioartificial pancreas.” The immunoisolation device or bioartificial pancreas can be commonly separated into two categories, intravascular and extravascular devices. The latter can further be divided into macroencapsulation and microencapsulation devices (Figure 2). Intravascular and extravascular classifications are based on whether or not it is connected directly to the blood circulation. The macroencapsulation and microencapsulation classifications depend on whether it contains one or more islets in the device.

#### 2.2.1. Intravascular Device

The intravascular device is designed to have a small chamber directly connected to the host's vascular system [44, 45]. Since the device is closely located to the blood supply, oxygen and nutrition diffuse into the device rapidly. The main biomaterial is the intravascular device is composed of copolymer polyacrylonitrile-polyvinyl chloride (PAN-PVC), which is similar to the material used in extravascular devices [46]. This kind of encapsulation system was initially used with autologous islet transplantation in the rodent model and normoglycemia was achieved for three months [47]. Furthermore, autologous islets in this device normalized the blood glucose in the monkey model. Although the modified versions of intravascular devices have been tested in allogeneic and xenogeneic transplant models [48, 49], such an encapsulation system has never been developed to the clinical level. The major concern that hampers the clinical application of the device is the development of thrombosis, which requires intensive anticoagulation treatment.

#### 2.2.2. Extravascular Device

The extravascular device is fabricated based on the principle of planar or tubular diffusion chambers. This type of device does not need anastomosis when it is implanted into the host and has an advantage over the intravascular device in terms of clinical application. The process of producing the extravascular device is called encapsulation.

The main advantage of macroencapsulation is the ease of implantation and the retrieval of the device. They may be implanted in the peritoneal cavity and in subcutaneous sites [50, 51]. On the other hand, one disadvantage of macroencapsulation that makes the device less applicable is the difficulty in the diffusion of nutrients and oxygen through the device, which tends to harm islets [45]. It has been reported that a tubular device made by copolymer PAN-PVC dramatically reduced adhesion and fibrosis, which has been observed in earlier studies. A set of encapsulated allogeneic islet transplantations in patients with T1DM was conducted using the more biocompatible tubular devices. The result showed that 2 weeks of graft survival was achieved without using any immunosuppressive medications [16]. However, this type of device was weak structurally and ruptured easily during implantation. Furthermore, the large number of islets required in the device leads the islets to clump together and undergo central necrosis. To overcome the drawback of the weak structure, different types of macroencapsulation systems have been proposed in the past [52–55]. A sheet type immunoisolation device made of alginate was reported by Storrs from Islet Sheet Medical [56]. This type of macroencapsulation device can be retrieved intact, which is an additional advantage in terms of clinical safety. Moreover, retrievability of the device allows for the quantitative assessment of islet viability and function. To overcome the problem of hypoxia and central necrosis of the implanted islets, a vascularization-enhanced macroencapsulation device was produced by TheraCyte [57]. This device is suitable for subcutaneous implantation and greatly beneficiated patients with T1DM. TheraCyte reported that islets encapsulated in such device survived for an extended period of time in a xenotransplanted animal model [58]. Most recently, another study using this device revealed that islet allografts were protected in immunized recipients [59].

The main advantages of the microencapsulation system over macroencapsulation are its stable mechanical structure, large surface area-to-volume ratio, and improved diffusion profile. Due to the flexible and adjustable characteristics, the microcapsules are mostly fabricated from hydrogels. Over the past 30 years, hydrogels including alginate [60], poly(hydroxyethyl methacrylate-methyl methacrylate), agarose [61], acrylonitrile copolymers, chitosan [62], and polyethylene glycol (PEG) [63] have been frequently used for microencapsulation. To date, the most preferable material for microencapsulation is alginate. The principle of making microcapsules is based on the envelopment of individual islets in a droplet, which is transformed into a rigid capsule by gelification (in the case of alginate beads) followed by polycation coating (in the case of multiple-layered microcapsules).

Alginate, a collective term for a family of polysaccharides synthesized by seaweed and bacteria, is used in a wide range of foods, pharmaceutical products, and other applications [64]. In molecular terms, alginates are binary linear polysaccharides composed of two monomers, *α*-L-guluronic (G) and *β*-D-mannuronic (M) acid, which form M blocks, G blocks, and blocks of alternating sequence (MG) [65]. In nature, alginates are found to exhibit great variations in composition and arrangement of the two monomers in a polymer chain. Blocks of repeating G units (G blocks) form cavities that bind divalent cations, which cross-link G blocks of other alginate chains [66]. This in turn allows for the formation of gels as capsules. Hence, G block sequences are required for the alginate to form a strong gel with divalent ions such as Ca^2+^, Ba^2+^, and Sr^2+^. A strong correlation therefore exists between the sequential structure and functional properties of alginates.

To increase the stability and to reduce the permeability of alginate gel beads, a polycation layer is traditionally added to the alginate gel core [67–70]. However, the successful use of alginate-polycation capsules as carriers for insulin producing cells *in vivo* has been hampered by the capsule's lack of biocompatibility as well as their mechanical instability. These disadvantages have made controlled insulin release and immunoprotection of islets difficult to achieve. The major obstacle for stability is swelling, causing an increase in pore size and ultimately breakage. This is caused by the loss of calcium from the calcium-alginate gel by, for example, phosphate and citrate, which can bind calcium, and nongelling ions such as sodium that over time will exchange some of the calcium in the gel [71].

### 2.3. Insulin Release Kinetics of Encapsulated Islets

**Figure 3:**
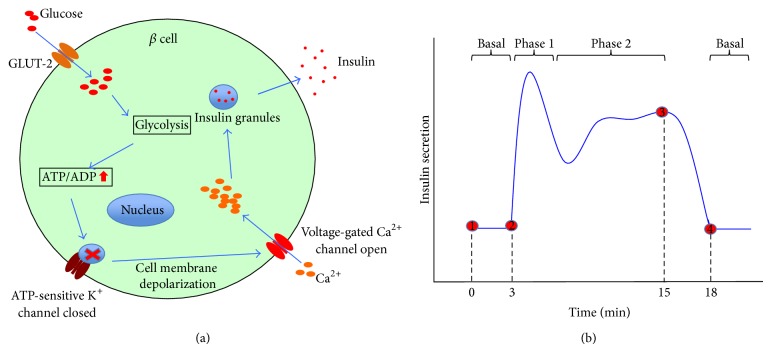
Diagram of insulin secretion from pancreatic *β* cells. (a) Cellular representation of an insulin-release process; (b) Graphical display of the biphasic insulin secretion.

Pancreatic *β* cells, which constitute 65–80% of the total cells in an islet, play a fundamental role in controlling metabolism through insulin secretion. Insulin release from *β* cells is controlled by the *β* cell's electrical activity, metabolic events, and ion signaling. These sets of intricate actions display the complex kinetic profile of biphasic and pulsatile responses to real-time changes in glucose levels [72, 73]. Insulin secretion is a complex and dynamic process. Glucose catabolism generates ATP through the mitochondrial Tricarboxylic Acid Cycle (TCA cycle), which consequently closes ATP-sensitive K^+^  (K_ATP_) channels, initiates plasma membrane depolarization, and increases Ca^2+^ concentration, through the rapid influx of Ca^2+^ via voltage-dependent calcium channels (VDCCs). This glucose-stimulated increase in Ca^2**+**^ concentration triggers the fusion of insulin granules with the cell membrane and the exocytosis of insulin, C-peptide, and proinsulin [74–77] (Figure 3(a)). Alternate pathways for insulin secretion, independent from K_ATP_ and Ca^2**+**^ concentrations, have been described [78, 79]. However, the K_ATP_ and Ca^2**+**^ concentration-mediated pathway remains the primary mechanism of glucose-stimulated insulin secretion. The normal response of *β* cells to glucose stimulation is the biphasic secretion process. The first phase corresponds to a transient and clear increase in the secretion rate. This is followed by a sharp decrease to the lowest secretion rate and a constantly flat or gradually increasing second phase that lasts as long as glucose stimulation is applied (Figure 3(b)). The secretion profiles, which are influenced by the environmental stimuli and controlled by the intrinsic characteristics of *β* cells, are thought to be important for insulin effects; however, the underlying mechanism of such dynamics has not been fully revealed [80, 81].

In physiological conditions, because of the rich blood supply to the pancreas, the *β* cells in islets detect hyperglycemia and release insulin rapidly to maintain glucose homeostasis. The transplanted naked islets lose direct connections to blood vessels, so diffusion is the only method for glucose and insulin to transport between the body and the islets. Regarding the transplanted encapsulated islets, the situation is presumably worse because molecules have to diffuse through the capsules. Therefore, it is of the upmost importance to understand the kinetics of insulin release from encapsulated islets.

The first known article regarding the kinetics of insulin release from encapsulated islets was published in 1988 by a research group from France. In this study glucose stimulated insulin release from islets, which were encapsulated in two different sized alginate-polylysine microcapsules (350 *μ*m and 650 *μ*m), were compared. The results showed that upon high glucose stimulation, the smaller microcapsules released a significantly higher amount of insulin compared to the larger microcapsules. However, the amount of insulin secreted from the smaller encapsulated islets was seven times less than that from naked islets [82]. In a recent study from 2009, the insulin release profile from encapsulated mouse insulinoma 6 (MIN6) cells was compared to that from nonencapsulated MIN6 cells. The kinetics of insulin release was more sluggish and the insulin release rate was lower in the encapsulated cells compared to the nonencapsulated cells [83]. Apparently, from the previously discussed studies, encapsulated islets or cells tend to show reduced insulin secretion when compared to nonencapsulated islets or cells. An interesting conclusion drawn from the combination of this study [83] and another study [84] is that the slowed insulin release was due to a delayed uptake of glucose through the semipermeable membrane, but not primarily due to a slowed release of insulin from the encapsulated islets. The aforementioned studies, therefore, imply that the challenge of optimizing the microencapsulation system is not only to make capsules of a reduced size, but also to adjust the permeability properties of the capsule pores in order to allow for the ease of diffusion of glucose molecules.

Recently, a microfluidic perifusion system has been introduced and developed in our research group. This system was designed to more precisely measure multiple key parameters that directly control *β*-cell insulin secretion and viability, such as mitochondrial electrical potentials, calcium influx, and insulin kinetics [85–87]. Most recently, this technology has been applied to evaluate microencapsulated islets. Our group has also developed a novel microfluidic-based cellular array capable of trapping individual microencapsulated islets in hydrodynamic traps. Using this device, we demonstrated high trapping efficacy for microencapsulated islets (~99%), with minimal physical stress on the cells (data not shown). The unique integration of an atmospheric component has also allowed the device to study impacts of hypoxia on microencapsulated islets.

### 2.4. Immunology and Biocompatibility

Immunology studies the host's defense mechanisms against invasion of foreign organisms, either living or non-living. The immune response is often divided into two categories, innate and acquired immune reaction. The innate immune response is nonspecific and exists in all individuals. It does not distinguish between different organisms and acts rapidly upon the exposure of foreign invaders. The innate immune reaction typically initiates with cellular mediators such as macrophages and neutrophils. The acquired immune reaction is specific and not actively present in all individuals. This specific immune response requires the recognition of a specific antigen by lymphocytes including T and B cells.

Biomaterials are not firmly considered as organisms [88]; however, implantation of biomaterials in a host triggers an immune reaction, which involves many components of the immune system. Biocompatibility is commonly defined as the ability of a biomaterial or other medical device to perform its function properly in a specific application with an appropriate response in the host [89, 90]. As indicated by Williams DF, “biocompatibility refers to the ability of a biomaterial to perform its desired function with respect to a medical therapy, without eliciting any undesirable local or systemic effects in the recipients or beneficiary of that therapy, but generating the most appropriate beneficial cellular or tissue response in that specific situation, and optimizing the clinically relevant performance of that therapy” [89]. The immunoisolation device is not constructed solely by material for the main structure and it also contains islet cells. Therefore, in order for the device to be biocompatible, the bioartificial pancreas must carry out its proper function and it must not harm the host. For an immunoisolation device, biocompatibility has been referred to as the degree of fibrosis after implantation into the host. Recently work has focused on the implantation of microcapsules in larger animals, primarily NHPs, to evaluate the biocompatibility of the microcapsules for clinical islet transplantation.

#### 2.4.1. Implantation of Empty Microcapsules

The purpose of immunoisolation is to avoid immune rejection from the host. However, the device itself can trigger inflammatory reactions and different immune reactions. All biomaterials elicit an immune response from the host; known as the foreign body reaction. The foreign body reaction is considered as a nonspecific immune response and the reaction occurs as soon as the foreign materials are introduced to the host. The mechanisms and processes of the foreign body reaction are described extensively in several articles [91–94]. Generally speaking, the full process of the foreign body reaction can be described chronologically in the following order.

- Surgical procedure introduces an injury. This triggers the initial inflammatory reaction to the biomaterials starting with the formation of a provisional matrix [95, 96].
- Proteins from blood and interstitial fluids are in direct contact and attach to the biomaterials. These proteins trigger the activation of the coagulation system, the complement proteins, and the platelets [97–99].
- As a result of the activation of inflammatory mediators, wound healing regulators, and other types of immune cell reactions, fibrotic tissue will form over the foreign materials. The main inflammatory and wound healing mediators involved in this fibrotic formation are TNF-*α* [100], IFN-*γ* [101], IL-6 [102], IL-8 [102], MCP-1 [102], macrophage inflammatory protein (MIP)-1*β* [102], IL-4 [100], IL-13 [103], IL-10 [100, 104, 105], transforming growth factor (TGF)-*β* [106], and platelet derived growth factor (PDGF) [107]. The main immune cells associated with fibrosis formation are: monocytes, macrophages, dendritic cells, and lymphocytes [108].

As noted earlier, alginate is the most commonly used material for islet microencapsulation. The biocompatibility of microcapsules has been tested with the implantation of empty microcapsules in numerous animal models. The peritoneal cavity has been selected as an optimal site for *in vivo* analysis of microencapsulated islet implantation, as this site can harbor a large volume of microcapsules [14]. Furthermore, this site is easily accessible during implantation and is relatively safe. It has been reported previously that empty microcapsules, composed of purified alginate, do not elicit any significant foreign body reaction after implantation into the peritoneal cavity of rodents [109, 110]. However, implantation of empty microcapsules into the portal vein of pigs provoke extensive pericapsular cellular overgrowth [111]. This result indicates that portal vein microcapsule transplantation is incompatible with the current alginate composition.

#### 2.4.2. Implantation of Microcapsules Containing Allogeneic Islets

The evaluation of the function of microencapsulated islets in large animals is a necessary transit point between scientific studies in rodents and its clinical application for humans. Allotransplantation in large animals has been performed to mimic clinical islet transplantation. Soon-Shiong et al. initially reported the long-term reversal of diabetes in dogs using microencapsulated islet allografts [112]. In this study, encapsulated canine islets, using alginate-PLL microcapsules, were transplanted into the peritoneal cavity at a dose of 15,000–20,000 IEQ/kg. Two years graft survival was achieved in recipients that received a single encapsulated islet transplant with a month of anti-inflammatory medication. Recently, allografts in alginate-PLL microcapsules were tested in the absence of antirejection medications in pigs but large-scale studies were not documented [113, 114]. In 2008, Wang et al. published work on the normalization of blood glucose levels in dogs for up to 214 days with a single transplantation of microencapsulated allogeneic islets without immunosuppressive medication [39]. In this study, an encapsulation system consisting of alginate, CaCl_2_, PMCG, cellulose sulfate (CS), and PLL was first introduced in this animal model. The amount of islets used in this study was 20,000–90,000 IEQ/kg, which is significantly higher than that in similar previous studies [112]. This study implies that more islets are needed to normalize blood glucose levels if immunosuppressive medications are not administrated after transplantation. Although the NHP is considered as an optimal allotransplantation model, little is published in terms of microencapsulated islet transplantation. In our previous study, we conducted allogeneic islet transplantations in baboons using the modified PMCG microcapsules. Two diabetic baboons were transplanted with an average of 16,475 IEQ/kg encapsulated islets (2-3 transplants) and neither baboon achieved normoglycemia after transplantation. Evenly distributed microcapsules were observed in the peritoneal cavity. Retrieved microcapsules at 4 weeks posttransplant were intact and free of cellular overgrowth around the microcapsules.

In summary, a great amount of encapsulated islets are required to normalize blood glucose levels in large animals.

#### 2.4.3. Implantation of Microcapsules Containing Xenogeneic Tissue

Due to the shortage of donor tissues for patients with T1DM, xenotransplantation has drawn the attention of research facilities. Most xenotransplantation uses microencapsulated porcine islets as donor tissue. Sun et al. found that microencapsulated porcine islets transplanted into spontaneously diabetic cynomolgus monkeys survived for 120–800 days with no immunosuppression [115]. Other groups have tested their encapsulated porcine islets in nondiabetic monkeys [41, 42]. It is notable that all of these transplanted porcine islets were encapsulated in alginate-polycation based microcapsules, which is a microcapsule with less antibody permeability.

In our previous study, human islets encapsulated in Ca^2+^/Ba^2+^-alginate microbeads were transplanted into the peritoneal cavity of a diabetic baboon at a dose of 36,000 IEQ/kg. After transplantation, decreased blood glucose and positive C-peptide production were observed up to 2 weeks. Adhesion and clumping of the microcapsules were observed during laparotomy at day 76 posttransplant. Microcapsules that were retrieved at this point presented with fibrotic overgrowth. Xenogeneic tissue can trigger a stronger immune mediated rejection compared to allogeneic tissue, which may explain islet graft dysfunction in this study. Antibody responses against the encapsulated islets were found 20–35 days posttransplant. Similar results were observed in the transplantation of microencapsulated human islets into the peritoneal cavity of diabetic cynomolgus monkey (unpublished data).

It has been reported that transplantation of macroencapsulated pig islets can reverse diabetes in primates for 6 months without immunosuppression [116]. Most recently, Veriter et al. have reported the result of subcutaneous transplantation of macroencapsuled pig islets coencapsulated with mesenchymal stem cells. In this study, a significant correction of glycated hemoglobin was achieved in diabetic primate model [117].

### 2.5. Local or Short-Term Immunomodulation

As mentioned earlier, a variety of natural and synthetic polymers have been used in islet encapsulation. However, inconsistency and poor long-term results have been a major limitation for clinical application. The graft failure is usually initiated by several factors including poor biocompatibility of the implanted materials, hypoxic conditions for islets inside of the capsules, and incomplete immunoprotection [118–120]. Thus, local or short-term immunomodulation and a nonsystematic immunosuppressive treatment have been investigated to improve the encapsulated islet transplant outcomes.

Biocompatibility of capsules is crucial for the long-term survival of the islet graft. It was demonstrated that a 10-day immunosuppressive medication regimen significantly reduced the fibrotic overgrowth around the intraportally implanted empty microcapsules [121]. Our group also tested the beneficial effects of 2-week long T-cell directed immunosuppressive medication and anti-inflammatory agents (TNF-*α* blocker) on the biocompatibility of Ca^2+^/Ba^2+^-alginate microbeads in cynomolgus monkeys. The results showed that the medications could only prevent fibrotic overgrowth on the surface of the implanted empty microbeads for as long as the medications were administered. This suggests that the extended use of immunosuppressants may have to be administrated to make the Ca^2+^/Ba^2+^-alginate microbeads biocompatible, which diminishes the goal of the encapsulation strategy (unpublished data).

Incomplete immunoprotection is mainly caused by the uncontrollable passage of proinflammatory cytokines and other immunoreactive molecules with low molecular weights, such as IL-1*β* (17.5 KD) and TNF-*α* (51 KD) through the biopolymer membrane [30, 122, 123].

Therefore, strategies to block those cytokines have been studied in recent years to improve the graft survival after encapsulated islet transplantation. In a recent study, a peptide inhibitor for the cell surface IL-1 receptor (IL-1R) was conjugated to the hydrogel for capsules to block the interaction between the immobilized cells and the cytokines [124]. In another strategy, Sertoli cells were used in co-encapsulation with islets cells. These cells are located in the convoluted seminiferous tubules of testes and have been shown to inhibit T-and B-cell proliferation and IL-2 production [125]. Cotransplantation of islets with Sertoli cells was shown to have varying protective effects on graft survival in allo- [126], concordant (rat to mouse) and discordant (fish to mouse) xeno- [127, 128], and autoimmune [129] transplant models. It was published that the Sertoli cells improve the functional performance of alginate-PLL microencapsulated islets in xenotransplant models (rat-mouse) [130]. However, this approach has not advanced significantly enough to be used in clinical trials.

### 2.6. Encapsulated Islet Transplantation in Patients with T1DM

Table 3 lists the clinical trials of encapsulated islets transplanted in patients with T1DM. Soon-Shiong et al. reported a successful human encapsulated islet transplant in a diabetic patient who was receiving immunosuppression for a functioning kidney graft [15]. In the study, a total of 15,000 IEQ/kg alginate-PLL encapsulated islets were implanted intraperitoneally. Insulin independence was demonstrated for 9 months after the procedure, with tight glycemic control noted.

Scharp et al. subcutaneously implanted a PAN-PVC macroencapsulation device containing allogeneic islets into 9 patients [16]. The results concluded that macroencapsulated human islets could survive at the subcutaneous site and that semipermeable membranes can be designed to protect against both allogeneic immune responses and the autoimmune reactions of patients with T1DM.

Calafiore et al. transplanted alginate-PLO microcapsulated islets in a human clinical trial without immunosuppression. In 2006, the results of the first two patients were published and both patients showed increased C-peptide serum levels, as a measure of islet graft function. Several weeks after transplantation, these two patients presented with an ephemeral incline in exogenous insulin consumption [17]. In 2011, the same group published the results of encapsulated islet transplantation in 4 patients, which included the follow-up results of the initial two patients reported in 2006 and two other patients transplanted afterwards [131]. So far, the results from 4 patients have been reported. In all cases the group observed no side effects of the grafting procedure, nor any evidence of immune sensitization. All patients exhibited a lower intake of exogenous insulin, approximately half of the pretransplantation consumption levels.

Tuch et al. transplanted allogeneic islets encapsulated in Ba^2+^-alginate microbeads into four diabetic patients without immunosuppression. C-peptide was present on day one after transplantation, but disappeared within a period of one to four weeks. In a recipient of multiple islet infusions, C-peptide was detected at 6 weeks after the third infusion and remained detectable for 30 months. Neither insulin requirement nor glycemic control were altered in any of the patients [18].

From 2005 to 2006, two companies, Amcyte, Inc., and Novocell, Inc. announced clinical trials involving encapsulated islet transplantation in patients with T1DM. Amcyte, Inc. planned to conduct clinical trials in twelve patients using islets encapsulated in alginate-PLL microcapsules. These microcapsules were further embedded into a macrocapsule for implantation. Another company, Novocell, Inc. (current name ViaCyte, Inc.), initiated phase 1/2 clinical trials of PEG-encapsulated islet allograft implantation in patients with T1DM. Twelve patients were enrolled in this clinical trial. However, this particular study was terminated. Currently, there is limited information available regarding these two clinical trials.

Xenotransplantation has attracted much attention in the field of islet transplantation. In the light of such consideration, transplantation of microencapsulated xenogeneic islets, especially porcine islets, has commenced in patients with T1DM. In 1996, Living Cell Technologies (LCT), a company based in New Zealand, initiated a clinical trial involving encapsulated porcine islet transplantation. In this trial, porcine islets were encapsulated in alginate-PLO microcapsules and implanted into the peritoneal cavity of patients without immunosuppression. Nine and a half years after transplantation, laparotomy of one of the patients showed the presence of microcapsules in the peritoneal cavity, some of which still contained live pig islet cells. However, the majority of cells appeared to be necrotic [132]. As of now, the company reported in their website that a total of 14 patients with T1DM were enrolled in the phase 1/2 clinical trial of DIABECEL conducted in New Zealand and Russia [43]. The first four patients received approximately 10,000 IEQ/kg encapsulated islets and showed an average reduction of 76% in episodes of clinically significant hypoglycemia unawareness after 30–52 weeks of followup. Four patients from each of the second and third groups received 15,000 and 20,000 IEQ/kg of encapsulated islets respectively and the followup of these particular patients is ongoing. The last two patients have received a dose at 5,000 IEQ/kg and were enrolled to construct the dose ranging data needed to determine a target product profile for phase 3 clinical trials. Based on the most recent newsletter from the website, a registration study has been launched in 2013 for phase 2b/3 clinical trials, in which 30 patients were enrolled. The LCT product, DIABECEL, is expected to be commercially available in 2016 [43].

Most recently, Jacobs-Tulleneers-Thevissen et al. published work on transplantation of Ca^2+^/Ba^2+^-alginate microbeads containing allogeneic islets in a patient [19]. The alginate microbeads were harvested 3 months after transplantation and were conglomerated in the peritoneal cavity. In another report, Sernova Corp announced a commercial product of the macroencapsulation device called the Cell Pouch System. This device can be subcutaneously implanted. The device has a unique ability of releasing antirejection drugs locally. The Cell Pouch System is currently preparing for clinical trials [133].

### 2.7. Alternative to Allogeneic Islets from Deceased Donors for Clinical Encapsulated Islet Transplantation

As mentioned before, in the ongoing clinical islet transplantation protocols, the donor pool cannot provide enough islets to treat all potential patients. Therefore, different cell sources have been investigated to overcome this problem, including xenogeneic pig islets [134–136], genetically engineered insulin-producing cells [137], and insulin-producing cells differentiated from stem cells [138–140]. Since these cell types are potential alternative cell sources for clinical islet transplantation, they are also being considered for clinical encapsulated islet transplantation. However, to date only encapsulated porcine islets have been tested in patients with T1DM [43]. The encapsulation of other cell types has only been tested in experimental animal models to investigate the features of growth, differentiation, and maturation [37, 141, 142].

## 3. The Present and Future

At present, there is a large amount of islet encapsulation-related research in progress around the world trying to eliminate the use of immunosuppressants in patients with T1DM. This research is largely uncoordinated and a well-documented systematic analysis of the various capsule types has not been completed. The correlation between NHPs and human subjects in biocompatibility of device and function of transplanted islets is poorly demonstrated. Despite the numerous clinical trials conducted by academic institutes and biotechnological companies, encapsulated islet transplantation has not been perfected [17–19, 43, 131]. With regard to the mixed set of results, there are three main factors limiting the progression of microencapsulated islet transplantation towards clinical application. First, the variability of raw materials in the manufacturing process has impeded the development of a reliable microencapsulation system. Second, current biocompatibility testing relies heavily on *in vivo* rodent models, which does not strongly support patients with T1DM. Finally, there is a significant inconsistency in results observed among individual laboratories even with the use of similar biomaterials and experimental approaches.

Taking all these obstacles into account, the development of a centralized *in vitro* and *in vivo* testing center in the future would allow for a more comprehensive, consistent, and species-specific examination of biocompatibility for the encapsulation system. A collaborative consortium may need to be organized, which should lead to the standardization in material selections, techniques, animal models, and procedures. Under active collaboration between research facilities, the end goal of providing islet encapsulation as a viable cure for patients with T1DM without immunosuppressant would be achievable.

## Figures and Tables

**Table 1:** Enzyme types used for human islet isolation.

Enzyme types	Manufacture	Concentration	Digestion time (min)
Sigma type V collagenase	Sigma	1 g/350 mL	14
BM Type P collagenase	Boehringer-Mannheim	0.7 g/350 mL	25
Liberase HI	Roche Applied Science	0.5 g/350 mL	16
Liberase MTF C/T, GMP grade	Roche Applied Science		19
Collagenase		0.5 g/350 mL	
Thermolysin		0.015 g/350 mL	
Collagenase NB 1 (premium and GMP)	SERVA Electrophoresis GmbH		14
Collagenase		1600–2286 PZ units/350 mL	
Neutral Protease		200–286 DMC units/350 mL	
VitaCyte C1 collagenase	VitaCyte		20
CIzyme collagenase HA		15–18 units/g tissue	
CIzyme thermolysin		1.25 DMC units/g tissue	

MTF: mammalian tissue free.

GMP: good manufacturing practice.

DMC: dimethylcasein.

**Table 2:** Product-Release Test for islets before transplantation.

Test	Test method	Criteria
Purity	DTZ staining is used for islet identity, which is visualized by qualified personnel	≥30%
Viability	Fluorescent dye (FDA and PI) staining is used for islet viability, which is determined by qualified personnel	≥70%
Islet yield	DTZ staining is used for islet identity and islet number is counted by qualified personnel	First transplant: ≥5,000 IEQ/kg RBW Second transplant: ≥10,000 IEQ/kg RBW
Transplant tissue volume	Centrifuge and measure packed cell volume in conical tube	≤10 mL
Microbiological test	Gram stain on 100 *μ*L smear with microscopic examination by qualified personnel	No intact organism observed
Endotoxin content	QCL-1000 Chromogenic LAL Test Kit, Cat number 50-647U, (BioWhittaker, Inc.)	≤5 EU/kg body weight of the potential recipient
Glucose static incubation	*In vitro* insulin release in 1.6 mM and 16.7 mM glucose. Expressed by SI	≥1.5

DTZ: dithizone.

FDA: fluorescein diacetate, for live cells.

PI: propidium iodide, for dead cells.

RBW: recipient body weight.

SI: stimulation index.

**Table 3:** Encapsulated islet transplantation in patients with T1DM.

Investigator or company	Type of encapsulation	Islet source (patient number)	Immunosuppression	Transplant site
Soon-Shiong et al. [15]	A-PLL microcapsule	Allogeneic (1)	Yes, after kidney transplantation	Peritoneal cavity
Scharp et al. [16]	PAN-PVC diffusion chamber	Allogeneic (9)	No	Subcutaneous site
Calafiore et al. [17]	A-PLO microcapsule	Allogeneic (4)	No	Peritoneal cavity
Tuch et al. [18]	Ba^2+^-alginate microbeads	Allogeneic (4)	No	Peritoneal cavity
Amcyte, Inc.	A-PLL microcapsule	Allogeneic (12 intended)	No	Peritoneal cavity
Novocell, Inc. (ViaCyte, Inc.)	PEG conformal coating	Allogeneic (12 intended)	No	Peritoneal cavity
Living Cell Technologies (LCT)	A-PLO microcapsule	Porcine insulin-producing cells (DIABECEL)	No	Peritoneal cavity
Jacobs-Tulleneers-Thevissen et al. [19]	Ca^2+^/Ba^2+^-alginate microbeads	Allogeneic (1)	No	Peritoneal cavity
Sernova Corp.	Macroencapsulation Cell Pouch System	Allogeneic (under preparation)	NR	Subcutaneous site

A-PLL: alginate-polylysine-alginate microcapsule.

PAN-PVC: polyacrylonitrile-polyvinyl chloride.

A-PLO: alginate-polyornitine-alginate microcapsule.

NR: not reported.

PEG: poly(ethylene glycol).
